# Book Reviews

**Published:** 1829-10

**Authors:** 


### CRITICAL ANALYSES.

Quæ laudanda forent, et quæ culpanda, vicissim

Illa, prius, cretâ; mox hæc, carbone, *notamus*.—Persius.

*The Influence of Climate in the Prevention and Cure of Chronic Diseases, more particularly of the Chest and Digestive Organs: comprising an Account of the principal Places resorted to by Invalids in England and the South of Europe; a comparative Estimate of their respective Merits in particular Diseases; and general Directions for Invalids while travelling and residing Abroad. With an Appendix, containing a Series of Tables on Climate.* By James Clark, m.d. Member of the Royal College of Physicians of London; corresponding Member of the Royal Medical Society of Marseilles, of the Medico-Chirurgical Society of Naples, of the Medical and Physical Society of Florence, of the Academy of Sciences of Sienna, &c. &c.—8vo. pp. 328. T. and G. Underwood, London, 1829.

The author of the present volume has already introduced himself very favorably to the notice of his professional brethren, by a brief, but very excellent, work on the Climate and Medical Institutions of France and Italy, which was published about nine years ago. Since that time he has had ample opportunities of observing the nature of the climate of the south of Europe, and its effects on disease. He has also made himself acquainted with the milder parts of England, with the view of ascertaining their respective merits, and of comparing them with the climates of the south.

The work is divided into two parts. In the first, Dr.

Clark has endeavoured to determine the general physical character of the milder climates of the south of Europe and of England; to point out the manner in which the climate of different places resorted to by invalids is modified by local circumstances; and to compare these places relatively to their influence on disease. In the second part, an account is given of the principal diseases which are benefited by a mild climate. The importance of consumption and disorders of the digestive organs, their extreme frequency in this country, and the close relation in which they stand to climate as a remedy, have induced Dr. Clark to comment upon them at some length. His inquiries have been directed chiefly to the causes and origin of consumption, with the view of establishing rules for its prevention; and he feels convinced that, by adopting the system of management from early infancy which he has laid down, that a great improvement might be effected in the general health of many among the higher and middle classes of society in this country, and that the children of delicate, and even of diseased parents, might, by proper care, be reared so as to overcome, in a large proportion of cases, their hereditary disposition to disease.

Part I. On Climate. It cannot be necessary to adduce any formal proofs to show that the influence of climate in the prevention and cure of diseases is, for many reasons, a subject of peculiar interest to the inhabitants of this country. The most careless observer must be aware that some of our most fatal diseases are to be attributed to the inclemency of our seasons, and that many others of great frequency, if they do not derive their origin immediately from our climate, are at least greatly aggravated by it. Change of climate, therefore, as a remedy, is very frequently had recourse to, both with and without the sanction of the physician. That even he sometimes abuses it, we cannot doubt; for we have frequently seen patients labouring under incurable disease, separated from all domestic comfort, and transported, at great expense and equal inconvenience, to a foreign clime, without the most remote possibility of advantage. Invalids who unadvisedly seek to recover health by the same means, not unfrequently rush into every irregularity, both moral and physical, from a ridiculous and too general opinion that, provided they do but change the scene and temperature, they must derive benefit, whatever imprudence they may commit. Upon this important subject Dr. Clark offers some very sensible remarks, which are introductory to his general subject. Neither travelling nor change of climate, nor the combined influence of both, can produce permanent benefit, unless directed with due regard to the nature of the case, and aided by proper regimen. The patient who goes abroad for the recovery of his health is cautioned not to expect too much from the mere change of climate. The air or climate is often regarded by invalids as possessing some specific power, by virtue of which it directly cures the disease. This is a very erroneous view of the matter, and not unfrequently proves the bane of the invalid, by leading him, in the fulness of his confidence in climate, to neglect other circumstances, an attention to which may be as essential to his recovery as even that in which all his hopes are placed. If the patient wishes to reap the full measure of good which his new position may place within his reach, he must trust more to himself, and to his own conduct, than to the simple influence of climate, however genial.

England. Before travelling beyond sea in search of a climate that may prove beneficial to his disease, the invalid will, or ought to, inquire what resources the limits of our own island afford. Dr. Clark believes that England possesses advantages which have not been made so fully available in this way as they might have been; and that many invalids, for want of discrimination in applying the proper climates to the diseases to which they are most suited, have gone abroad in search of that which they might have found almost at their own doors. We admit the truth of this observation; but it must be remembered that the novelty of the scene which is presented in a foreign country to an untravelled Englishman produces an agreeable mental excitement, which is very frequently productive of the best effects upon the corporeal malady, and which is altogether independent of mere change of climate. We have known many invalids, who have suffered more perhaps from exhaustion of mind arising from close application to worldly affairs at home than from bodily disease, who have in vain visited some of the most delightful and salubrious parts of their own country, but have been quickly restored by a trip to foreign parts.

*London.* The mean annual temperature of our metropolis is 50° 39, being one and a half degree above that of the environs, with the exception perhaps of the warmer parts of Brompton and Chelsea, which are peculiarly sheltered.

"This difference of temperature between the metropolis and surrounding country, as the physician ought to know, is very unequally distributed throughout the year and throughout the day.

The excess of the city temperature arrives at its maximum in January, at which time it exceeds that of the environs by three degrees; but the difference throughout the whole of the winter is less than it is in the summer. In the spring months, the temperature of the environs becomes nearly equal to that of London, and in the month of May it rather exceeds it." (P. 18.)

*South Coast of England.* The minimum temperature observed on the south coast is, generally, from three to four degrees above the minimum temperature of London. Nor is the temperature of the south coast subject to the same extent of range as that of London and the interior.

"In steadiness of climate, as deduced from the variation of temperature between successive days, the south coast does not appear to possess any very remarkable superiority over London itself. Of the places on this tract of coast which have been particularly examined, Southampton is the most variable in its temperature, equalling in this respect the environs of London." (P. 22.)

A greater quantity of rain falls on the south coast than at London, the ratio being, as nearly as could be ascertained, as thirty to twenty-five. The general character of the climate of this district is "humid and heavy," and many parts of it are subject to aguish complaints. Chichester the author considers the best winter residence for that class of invalids likely to be benefited by a climate of this kind.

*Hastings*, from its low situation, and the height of the neighbouring cliffs, is protected in a considerable degree from the north and north-easterly winds. "One of the principal disadvantages of Hastings is its confined situation, by which its climate is limited to a small local extent, owing to the close manner in which it is hemmed in on the sea by the steep and high cliffs which rise immediately behind it." In autumn, and even December, the climate of Brighton is warmer and more steady than that of Hastings. According to the experience of Dr. Clark, the latter place is unfavorable in nervous complaints, to persons subject to headach, and to languid or relaxed habits. For those who have suffered from ague, Hastings is a doubtful residence.

*Brighton.* Here the climate is, in respect of closeness, the reverse of Hastings. It is dry, elastic, and bracing, and, to persons of nervous or relaxed habits who can bear cold winds, it is much more congenial. The most favorable season of the year at Brighton is the autumn and beginning of winter. The early part of the spring is the worst season.

*Isle of Wight.*—The only part of this island well adapted for the winter residence of invalids is *Undercliff.* In this district the scenery is splendid; there are no moist or impure exhalations, and it is completely sheltered from the north, north-east, north-west, and west winds. Snow is rarely seen, and frosts are but partially felt.

"The mildness of the climate, the dry nature of the soil of Undercliff, and the extent to which it is sheltered from cold winds, by affording ample space for exercise, are great advantages to invalids threatened with pulmonary disease, or others to whom exercise in the open air, in a mild climate, is desirable. In this respect it is probably superior to any place in this line of coast. The principal objections to it as a residence for invalids, are the scantiness of accommodations, and its distance from medical advice. On this account it is, perhaps, at present rather calculated for the retreat of the valetudinarian, who does not stand in need of much medical attendance, than for the real invalid." (P. 29.)

*South-western Coast.* The south coast of Devon, the warmest part of this district, has a winter temperature nearly two degrees higher than that of the coast of Sussex and Hampshire, and from three to four warmer than that of London.

"During the months of November, December, and January, the difference is most remarkable; amounting on the average, in the sheltered places, to five degrees above London. In February, the difference falls to three degrees; and, in March and April, the excess of the mean temperature over that of London does not amount to one degree. It ought also to be remarked that this difference takes place principally in the night; as the difference between the lower extremes of London and the south-west coast is to the difference of the higher extremes as four to three; a less disproportion, however, than occurs between the south coast and London. Hence, when compared with the latter, the days are proportionally warmer on the south-western than on the southern coast; whilst the nights at both places are nearly equal." (P. 30.)

*Torquay.* The general character of the climate of this coast is soft and humid. It is remarkably protected from north-east winds, and also from the north-west.

Of the places on this coast frequented by invalids, *Dawlish* perhaps deserves the preference after Torquay. *Exmouth* is well sheltered, but exposed to damp and fogs.

*Sidmouth* possesses little of the characteristic qualities of this climate. It is inferior to all the other places as a winter or spring residence. As a watering place in summer or autumn, it may be agreeable.

"The climate of the coast of Devonshire is found very beneficial in various forms of disease. I have known it serviceable in chronic affections of the throat, trachea, and bronchia, proceeding from irritation, or a low degree of inflammation of these parts, and attended with a dry cough, or with little expectoration; likewise in an irritable or morbidly sensitive state of the stomach, and in hypochondriacal affections, the consequence of such a state. In dysmenorrhœa, and all nervous sympathetic affections dependent on that disorder; in a highly sensitive state of the nervous system, and in most diseases of general irritation, advantage may be expected from this climate. On the other hand, it certainly exerts an unfavorable influence on nervous headachs, and on all nervous complaints arising from relaxation or want of tone of the nervous system; it is injurious also in pure dyspepsia, when the tone and sensibility of the stomach are below par, as indicated by pale lips, a pale clammy state of the tongue, and languid circulation; and it will be found no less unfavorable in menorrhagia, leucorrhœa, and all diseases accompanied with much general relaxation of the system, or with much discharge from the affected organs." (P. 34.)

What may be the real estimation in which the climate ought to be held in consumptive complaints, Dr. C. has much difficulty in saying. But, as the invalid will be exposed to less rigorous cold, and for a shorter season, will have more fine weather, and consequently more exercise in the open air, he gives himself a better chance by passing the winter here, than he could have in any more northern part of our island.

*Cornwall.* There are several places on the north and south coasts of the extended Cornish peninsula that deserve attention; but the imperfection of our meteorological data greatly circumscribe our investigations. The only places, therefore, of which the author is enabled to speak with some confidence, are one or two near the south-west extremity of the peninsula.

*Penzance* can hardly be said to be sheltered from any wind. The climate here is peculiar, and unlike any other which Dr. C. has met with. The mean annual temperature is only 1°.77 above that of London. "But this temperature is very differently distributed over the year at the two places. Although Penzance is only a degree and a half warmer than London for the whole year, it is 5½° warmer in winter. It is 2° colder in summer; scarcely 1° warmer in spring; and only about 2½° warmer in the autumn." Penzance is, on an average, nearly 6½° warmer than London during the night in winter, and little more than 3° warmer during the day. For equality of temperature throughout the day or year, Madeira is the only climate the author has examined which is superior to Penzance. In the spring this latter place loses its superiority of climate.

"In the other elements of climate, this district has less peculiar advantages. There falls at Penzance nearly twice as much rain as at London, the annual average at the former place being forty-four, and at the latter only twenty-five inches. We have reason to believe also that the number of days in which rain falls is greater at Penzance than at London, although this is not the result of Mr. Giddy's observations, as given in his tables." (P. 42.)

Another disadvantage of the climate of the south-western extremity of our island, is its liability to violent and frequent storms of wind; and of this disadvantage Penzance appears to partake largely.

The general qualities of *Falmouth* are nearly the same as those of Penzance.

"With respect to the effects of the climate of the Land's-end on disease, the disadvantages which attach to it generally, in point of humidity and exposure to winds, are such as in a great measure to neutralize the superiority which it possesses over the other climates of England in mildness and equability of temperature. In its general characters, this climate resembles so closely that of the south coast of Devonshire, that the remarks formerly made on the influence of the latter on disease apply nearly to it. Regarding its influence on consumption, we have the testimony of Dr. Forbes, founded on ample experience, that little is to be expected from it; but we ought to admit, at the same time, that in this respect it but shares the opprobrium with every other climate, in the advanced stages of that disease." (P. 47.)

Dr. Forbes states that, in many cases of chronic bronchitis, simulating phthisis, the health was greatly improved, and in some completely restored, from a state of great debility and seeming danger, by a residence at Penzance.

"The consumptive cases in which the soft humid atmosphere of this place is likely to prove beneficial, are those in which the disease is accompanied with an irritated state of the mucous membrane of the lungs, producing a dry cough, or one with little expectoration.

"In idiopathic tracheal and bronchial diseases of the same character, whether complicated with asthma or otherwise, and also in certain pure cases of the latter disease, it is likely to be very beneficial. When, on the contrary, there exists a relaxed state of the general system, or a disposition to copious secretion from the bronchial membrane, whether idiopathic or symptomatic of a tuberculous state of the lungs, or where hæmoptysis has occurred, I believe the climate of the Land's-end will generally prove injurious." (P. 48.)

*West of England.*

"The mean temperature of the western group of climates during the winter, is rather lower than that of the southern coast, but in

March and April rises rather above it. The mean annual temperature of Cheltenham appears to be about one degree warmer than London; its winter, spring, and summer, from one to two degrees warmer; but its autumn somewhat colder. Bath and Bristol, during the months of November and December, are nearly three degrees warmer than London. In January and February they do not average one degree warmer. In March, Bath and Cheltenham are rather colder than London; but Bristol continues from one to two degrees warmer during March, as well as April." (P. 51.)

On comparing Penzance with this tract, we find only one degree of difference in the mean annual temperature: in winter, however, Penzance is four degrees warmer; but in the spring and summer it is somewhat colder. In steadiness of temperature from day to day, this district offers very little advantage over London. "Great Britain," says Dr. Chisholm, "presents almost the extreme of irregularity in her climate; and probably the western counties are the most distinguished for that versatility, that capriciousness of temperature, so peculiar to the whole of England."* In this tract of country, Bristol and Clifton appear to afford the most eligible winter and spring residence for invalids. At the bottom of the hill, in the neighbourhood of the Bristol Hotwells, the most sheltered situations are to be found. One advantage it possesses over even the boasted coast of Devonshire, being free from dampness and mizzling rains. Dr. Chisholm is of opinion that the subincumbent limestone contributes much to the salubrity of the atmosphere. The absence of every thing like marsh must certainly contribute greatly to the purity and wholesomeness of the Clifton air.

"From all these testimonies in favor of the climate of the more sheltered parts about Bristol and Clifton, there appears sufficient evidence of this spot being the mildest winter, and more especially spring, residence in the west of England. This results also from its sheltered situation, and the evidence afforded by our meteorological registers, which, we have seen, make Bristol warmer than the south coast, and equalling that of Devonshire during the spring months. This country affords also a good summer climate; so that for invalids to whom the air of this district is suitable, it presents altogether one of the best residences throughout the whole year in our island." (P. 55.)

Having given an account of the warmer situations in England, Dr. C. proceeds to point out what are the advantages they offer generally to invalids, and what are the

* On the Statistical Pathology of Bristol and Clifton, by the late Dr. Chisholm. (Edinb. Med. and Surg. Journal, vol. xiii. 1817.)

diseases in which they are respectively beneficial. The whole of these places are considerably warmer during the winter and spring than England generally, and much warmer than the colder parts of it.

"But it must be kept in mind that, as has been before observed, there are other circumstances connected with the adaptation of climate to disease, which require attention as well as temperature. The particular nature of the disease and of the patient's constitution, and the character of the climate most suitable for these, will naturally be the first objects of the physician's consideration; but the nature of the climate in which the invalid has lived ought also to be taken into account. This last circumstance, namely, the comparative influence of any particular climate on different individuals, depending on the nature of that which they previously inhabited, has not, I believe, been sufficiently attended to. It deserves, however, the especial consideration of physicians when selecting a climate for their patients." (P. 58.)

For example, an inhabitant of one of the coldest parts of our island would feel the influence of the climate of the south-west of England (as far, at least, as regards temperature,) as much as an inhabitant of the latter would that of the south of Europe. It is, with few exceptions only, as a means of preventing the occurrence of tubercular disease in the lungs, when threatened, or of checking its progress in its early stages, that much benefit can be expected from any climate.

"In diseases depending upon, or connected with, much general or local irritation; in chronic inflammatory affections of the throat, trachea, and bronchia, accompanied with little secretion or expectoration; in indigestion, arising from a heated and irritated state of the stomach, and in the nervous and hypochondriacal affections originating in such a state; in dysmenorrhœa, and in dry irritable cutaneous diseases, the coast of Devonshire affords the most favorable winter climate. In cases of the kind referred to, in which it is desirable that the invalid should remain stationary during the whole year, the Land's-end would perhaps be preferable to the coast of Devon." (P. 59.)

In chronic diseases of the trachea and bronchia, attended with copious expectoration and dyspnœa; in dyspeptic disorders of a more purely nervous character, with a relaxed system, or a tendency to mucous or sanguineous discharges, the climates of the south-west of England and the Land's-end are unfavorable. For such cases, perhaps, Brighton will be found the "most favorable residence during the autumn and early part of the winter." The latter part of the winter, and still more the spring, at

Brighton is cold, as it is unprotected from the north-east winds.

FRANCE. The south of France has been long held in estimation for the mildness of its climate; and various parts of it, says the author,

"have been, and are still, annually resorted to by invalids from this country; although, I fear, without much discrimination, either as regards the qualities of the climate, or the nature of the diseases in which this is most likely to prove beneficial.

"The climate of the southern provinces of France admit of being classed under two divisions, namely, the south-eastern and south-western. These two regions differ essentially from each other in the physical characters of their climates: the latter resembles in its general qualities the south-western parts of England, the former is of a totally different nature. In their influence on disease, they differ also in a very remarkable manner; and unless the distinctive characters of each in this respect be kept in view by the physician, in selecting a residence in this country for invalids, great errors must be committed." (P. 63.)

*The West and South-west of France.* Under this title Dr. C. includes the whole tract of country from Brittany to Bayonne, comprising L'Orient, Nantes, La Rochelle, Bourdeaux, Montauban, Pau, and Toulouse. The climate of this part of France is directly opposed in its qualities to that of the south-east of France.

"Though, on the whole, less warm than the latter, its temperature is more equal, and the range of this less extensive, as well through the whole year, as through the period of day and night. It is, however, more changeable from day to day, and the changes themselves are very considerable. The mean annual temperature of the south-west of France generally, is about fifty-five degrees. This makes it six degrees higher than England generally, and four degrees higher than the south-west of England; but three degrees below the south-east of France, and four degrees below Italy. The days are not so fine as in the south-eastern parts of the kingdom, but the nights are not so cold in relation to the days. The climate may be characterized as soft, relaxing, and rather wet. Hence it is suitable for complaints to which the south-east of France is injurious, particularly gastritic dyspepsia, (or dyspepsia depending on an inflammatory state of the stomach,) and the dry bronchial irritations. In that class of consumptive patients, therefore, in whom the disease is complicated with either or both of these affections, and in whom, consequently, there is a great susceptibility to the influence of dry, keen winds, this climate will generally agree. Laennec found the southern coast of Brittany very favorable to consumptive patients; and he also observed that the proportion of consumptive diseases in this part of France was comparatively small." (P. 64.)

"Generally speaking, the climate of the south-west of France will be useful in chronic inflammatory affections of the mucous membranes accompanied with little secretion, as in chronic bronchitis not attended by much expectoration, or difficulty of breathing, and in similar morbid states of the larynx and trachea. It will be equally proper in dry scaly eruptions of the skin; in dysmenorrhœa; in certain kinds of headach, especially those induced or exasperated by sharp north-east winds; and in high morbid sensibility in general, when accompanied with that habit of body which the ancients called *strictum*. On the other hand, the same diseases occurring in relaxed habits in which there is a disposition to copious secretion, will be increased by this climate. Those who do not find inconvenience from the south-west winds of Devonshire will find the climate agreeable." (P. 65.)

*Pau* is the only place in this district of which it is considered necessary to give a particular account. It is upon the whole healthy. "Scrofula is rare, and consumption not a common disease." The mildness of the spring, and its little liability to winds, render this place favorable to chronic affections of the larynx, trachea, and bronchia. "In gastritic dyspepsia, also, Dr. Playfair has found it beneficial; and he has seen it useful in a few cases of asthma." Upon the whole, Pau appears to be one of the most desirable winter residences in the south-west of France for invalids labouring under chronic affections of the mucous membranes.

"Invalids labouring under, or liable to, attacks of rheumatism, should of course avoid Pau. In bronchial diseases, also, when accompanied with much general relaxation of the system, and with copious expectoration and dyspnœa, the climate will not in general prove beneficial; and Dr. Playfair considers it too changeable in consumptive diseases." (P. 72.)

Invalids who mean to winter at Pau should arrive there in the end of September, or early in October.

*South-east of France.* Various places in this district have been recommended as a good winter climate for consumptive patients,

"but nothing can be more unaccountable than how such an advice ever came to be given; as the experience of later years is in complete opposition to it, and the general and leading characters of the climate show that there never was the least reason to sanction it. That the country which has always been infested by the terrible *Circius* should have been chosen for the residence of the delicate and sensitive sufferer from pulmonary disease, is a striking proof of the very loose observations upon which medical opinions respecting climate have been formed. How this practice of sending consumptive invalids to the south-east of France originated, it is not of importance to inquire: that it is founded on error, I think I shall be able to prove, by a reference to the physical characters of the climate, the actual prevalence of consumption among the inhabitants, and, I may add, the total want of success which has attended the measure." (P. 73.)

The mean annual temperature of Provence generally is 58°. Dryness is one of the most remarkable characters of its climate.

"The general character of the climate of the south-east of France, therefore, is dry, hot, harsh, and irritating. Absolutely warmer than our own island and the south-western parts of France, its temperature is distributed through the year and through the day with great irregularity. It has a much wider range of temperature than our own climate; this being, when compared to that of England, as three to one for the year, and as two to one for the day. Sometimes the winter is very rigorous." (P. 75.)

The temperature is steadier than our own from day to day, but its changes, though less frequent, are more sudden and extensive.

"Although decidedly improper for consumptive patients, and for those labouring under irritation of the mucous membranes of the digestive or pulmonary organs, more especially irritation of the stomach, larynx, or trachea, this climate may prove useful to invalids of a different class. On persons of a torpid or relaxed habit of body, and of a gloomy, desponding cast of mind, with whom a moist relaxing atmosphere disagrees, the keen, bracing, dry air of Provence, and its brilliant skies, will often produce a beneficial effect. In some cases of chronic intermittent fevers, also, it proves very favorable." (P. 76.)

*Montpelier*. The climate of this place little deserves the reputation which it still enjoys as a residence for the consumptive. Phthisis is here a very common and a very fatal disease among the inhabitants: whole families are frequently destroyed by it.

*Marseilles* "is but little entitled to claim any exception from the general character of the climate of Provence." It is dry, variable, and subject to cold irritating winds, which are peculiarly injurious to consumptive patients. Marseilles is one of the towns in France in which phthisis is most prevalent. "Invalids requiring a dry climate, and capable of bearing keen, cold winds, will be benefited by a residence at Marseilles: patients labouring under intermittent fevers often get rid of them without medicine, on coming to this place." (P. 79.)

*Aix* cannot be recommended as a winter residence for the consumptive.

*Hyeres* is agreeably situated near the shores of the Mediterranean, "and is the least exceptionable residence for the pulmonary invalid in Provence."

*Nice* is nine degrees warmer in its mean annual temperature than London. "The range of temperature for the day is also less at Nice than at any part of the south of Europe; and in steadiness of temperature it ranks next to Madeira." During March and April, sharp easterly winds prevail here. The proportions of deaths in the hospital from consumption is said to be about one seventh of the whole mortality.

"When this disease (consumption) is complicated with an inflammatory or highly irritable state of the mucous membranes of the larynx, trachea, or bronchia, or of the stomach, Nice is decidedly an unfavorable climate; and, without extreme care on the part of such patients, and a very strict regimen, the complaint will in all probability be aggravated by a residence here. Indeed, the cases of consumption which ought to be sent to Nice are of rare occurrence. If there are any such, it is when the disease exists in torpid habits, of little susceptibility, or not much disposed to irritation; and when it is free from the complications which have been just mentioned. Even the propriety of selecting Nice as a residence for persons merely threatened with consumption, will depend much upon the constitution of the individual." (P. 89.)

Young persons threatened with phthisis have sometimes been benefited by residing here for one or two winters. In chronic bronchial diseases very salutary effects are produced by a residence at this place.

"The particular kind of bronchial disease most benefited by a residence at Nice, is that accompanied with copious expectoration, whether complicated with asthma (*humoral asthma*) or otherwise; and in the chronic catarrh of aged people it is particularly beneficial. This variety of bronchial disease is directly the reverse of that which is benefited by the south-west of France and of England; and I think it important here to remark, that, unless the distinctions which I have pointed out in bronchial diseases, and their complications, are attended to, great errors must be committed in selecting a residence for such patients." (P. 91.)

In most cases, the gouty invalid may here escape his usual winter attack, and perhaps return to his own country with improved health. In chronic rheumatism, scrofulous complaints, dyspepsia, and hypochondriasis, Nice is beneficial. But here, again, distinction is necessary.

"The cases of dyspepsia most benefited are those accompanied with a torpid, relaxed state of the system, with little epigastric tenderness, or any of those symptoms which denote an inflamed or very irritable state of the mucous membrane of the stomach. Where the latter state is the cause of the dyspeptic symptoms, Nice will decidedly disagree: indeed, as I have already observed, a degree of this affection is also endemic there." (P. 92.)

Where there is great relaxation and torpor of the constitution, the climate of Nice is extremely useful. It may be considered generally as "warm, exhilarating, and exciting, but, upon the whole, irritating; at least, to highly sensitive constitutions."

*Villa Franca* is more protected than Nice from the north and north-west winds, but it is open to the whole range of easterly winds, which are the most prevalent of the spring winds, and the most injurious to the invalid. Here there are but few accommodations for patients.

Italy possesses great diversity of climate, but Dr. Clark's observations are limited to that tract which is situated between the northern shores of the Mediterranean and the southern base of the Apennines.

"The climate which prevails over the whole of this region, while it exhibits a great similarity of character, differs in several respects from any of the climates already noticed: it is considerably warmer and less humid, but subject to a greater range of temperature, than that of the south-west of France; it is softer, less dry, and less harsh and irritating than that of Provence; suffering more from the heavy oppressive winds of the south, and less from the dry searching winds of the north." (P. 97.)

*Genoa* "is an unsuitable residence for invalids generally, nor is there much in the character of the climate to recommend it." The distribution of heat throughout the year is unequal, and the temperature by no means steady from day to day. "The climate is on the whole dry and healthy, but not suitable to delicate sensitive invalids. It is more congenial to relaxed, phlegmatic habits." For pulmonary affections Genoa is an improper residence: tubercular consumption is prevalent.

*Florence*, "though one of the most agreeable residences in Italy, is far from being a favorable climate for invalids, and least of all for those disposed to consumption." Fogs are more common here than at most parts of southern Italy. The winter is four degrees warmer than London, and nearly of the same temperature as at Penzance. Dr. Clark does not know any class of invalids for whom Florence offers an advisable residence.

"My own opinion, founded partly on observation, and partly on the reports of invalids, perfectly accords with that of Dr. Seymour, of London, and Dr. Down, of Southampton, whose more extensive opportunities of observation during a long residence and extensive practice at Florence make their testimony of greater value." (P. 101.)

It is one of the places in Italy which agrees least with children.

*Pisa* has long had the reputation of being one of the mildest and most favorable climates in Italy for consumptive patients. In winter it is seven degrees warmer than London, and two warmer than Penzance. In spring it is eight degrees warmer than London, and about seven warmer than Penzance. The range of temperature between day and night is very considerable.

"For invalids who are almost confined to the house, or whose power of taking exercise is much limited, Pisa offers advantages over either Rome or Nice: the Lung' Arno affords a warm site for their residence, as well as a sheltered terrace for their walks. But they must be careful to confine themselves to it; they should not venture into the cross streets before April." (P. 103.)

Cataract and ophthalmia are common, but this is the case over the whole southern parts of Italy. Calculous diseases are so rare that Vacca, during thirty-two years he had been operating on such patients from all parts of Italy has not had occasion to operate on one at Pisan.

*Naples* in climate resembles Nice. It is unfit for consumptive patients.

"Naples is, however, well suited as a winter residence for those who are labouring under general debility and derangement of the constitution without any marked local disease. The beauty of its situation, the brilliancy of its skies, and the interest excited by the surrounding scenery, render it a very desirable and very delightful winter residence for those who rather require mental amusement and recreation for the restoration of their general health, than medical treatment for any particular disease." (P. 105.)

*Rome* has a mild and soft, but rather relaxing and oppressive, climate. Its mean annual temperature is ten degrees higher than that of London. One peculiarity of it is the stillness of the atmosphere; and this quality of calmness is valuable in a winter climate for pulmonary diseases; more especially for diseases of the larynx, trachea, and bronchia. Among the more prevalent diseases of Rome, *malaria* fevers are the most remarkable; and upon this very important subject Dr. Clark offers many remarks, which the traveller will do well to attend to. He considers the malaria fevers of Rome to be of exactly the same nature, both in their origin and general characters, as the fevers which are so common in the fens of Lincolnshire and Essex, in Holland, and probably in certain districts over the whole globe.

"Though the term malaria, which was for a certain time restricted to the fevers of Rome, but which has now become almost a generic name for these diseases, has given rise to some confusion on the subject, even among medical men; the form and aspect under which these fevers appear, may differ according to the concentration of the cause, or to some peculiar circumstances in the nature of the climate or season in which they occur; but it is the same disease, from the fens of Lincolnshire and the swamps of Walcheren, to the pestilential shores of Africa; only increased in severity, *cæteris paribus*, as the temperature of the climate increases. In England and in Holland, these fevers generally appear in the simple intermitting form; often, but more rarely, in the remitting form; and they are, for the most part, easy of cure. In France, especially towards the south, the same fevers often assume a more formidable character. Those which, from their unusual severity, and the peculiar character of their symptoms, have received the name of *pernicious*, are by no means uncommon in the south-west of France; and in the rice districts of Lombardy, they are met with in all their varieties, and with a degree of severity perhaps equal to the more aggravated forms of the malaria fevers of Rome." (P. 112.)

These fevers have generally been attributed to the direct action of something exhaled from the soil: but of the nature of this agent we are quite ignorant, and its existence is even doubted by many. At Rome, malaria fever seldom appears before July, and ceases about October. "An idea prevails that full living and a liberal allowance of wine are necessary to preserve health in situations subject to malaria." This is an erroneous opinion. Dr. Clark has known many persons suffer in Italy from acting on it. The same stimulating diet which might be borne, and even prove useful, in the damp chilly atmosphere of Holland, is not suited to the exciting climate of Italy.

"Among the diseases benefited by a residence at Rome, I may rank Consumption. In the early stages of this affection, I have generally found the climate favorable. I have frequently known patients who had left England labouring under symptoms that gave much and just alarm, (such as cough, expectoration, &c.,) which continued during the whole journey, and entirely disappeared after a short residence in Rome. The same persons have remained comparatively free from all bad symptoms during the whole season; and this when, from the ultimate result of the case, there could be little or no doubt of the existence of tubercles in the lungs at the time. In the advanced periods of consumption, I cannot say that the climate proved of any benefit, the disease generally proceeding in the usual course, and perhaps even more rapidly (especially during the spring months) than it would have done in England. In some cases the disease was increased in a remarkable manner during the journey to Italy." (P. 128.)

The climate of Rome is generally beneficial in bronchial diseases; but, if the disease is accompanied with copious expectoration, and is without much gastric irritation, Nice is to be preferred. October is the period at which the invalid should arrive at Rome.

Of a Summer Residence. As a general rule, the summer climate of Italy will disagree with all invalids labouring under general debility and relaxation of the system, or an irritable state of the mucous membranes, or who are disposed to diseases of the nervous system; and when symptomatic fever, with morning perspiration, has shown itself, this, the author considers, will afford a still stronger reason against a summer residence south of the Alps, whatever may be the disease.

In the vicinity of Naples are several beautiful situations, much preferable to the town itself as summer residences. "The *Vomero* and the *Capo di Monte* afford some good stations close to the city; and, of the more distant ones, *Sorento* and *Castlemare* are the best." The island of *Ischia* is also resorted to as a summer residence, and it may deserve a preference by some individuals, on account of its mineral waters. The baths of this place are very useful in chronic rheumatism, chronic affections of the periosteum, in the cachexia of pseudo-syphilis, in local paralytic affections, and in obstinate cutaneous diseases.

*Sienna* is an unfavorable climate at all seasons for persons disposed to, or labouring under, pulmonary disease. For nervous, relaxed people, it forms a better summer residence than either Naples or the baths of Lucca.

*Switzerland.* As a summer residence,

"Switzerland, in point of convenience, certainly affords one very eligible; but much caution and prudence are required on the part of invalids labouring under pulmonary affections who remain there. The alternations of temperature in Switzerland are often very rapid and very considerable. The difference between the day and night is great, and there is often a sharpness in the air which proves irritating to sensitive invalids." (P. 144.)

The borders of the lake of Geneva afford, the author believes, the best situations for a summer residence in Switzerland; and the neighbourhood of Geneva is altogether the least exceptionable.

"For the consumptive invalid, whose symptoms already indicate a tuberculous state of the lungs, and to whom it is of the utmost importance to avoid congestion of these organs and irritation and inflammation of their mucous surfaces, no part of Switzerland affords, I believe, a very favorable climate." (P. 146.)

"The subjects of pulmonary affections, who have spent the summer in Switzerland, will do well to try the '*cure de raisins.*' Of the salutary effects of ripe grapes, taken in considerable quantity for some time, there can be no question. In irritation of the mucous membrane of the lungs and digestive organs, and in congestive states of the abdominal viscera, with a disposition to hemorrhoids, ripe grapes taken for some weeks in the quantity of several pounds a day, with a light diet and abstinence from wine and every thing exciting, will often prove very beneficial. On this subject the invalid will, of course, be directed by a physician on the spot." (P. 148.)

In closing his remarks on the choice of a summer residence, Dr. Clark particularly directs our attention to certain cautions which the invalid should not fail to bear in mind. Unless a journey in hot weather is conducted with great circumspection, the irritation and excitement arising from it in susceptible systems (especially where any organ is in a state of chronic inflammation, however slight in degree,) will do more mischief than any advantage that can be derived from a short residence in the best climate, or from the use of the most valuable mineral waters.

*Madeira* "has been long held in high estimation for the mildness and equability of its climate, and we shall find on comparing this with the climates of the most favored situations on the continent of Europe, that its character is well founded." It is almost free from keen cold winds, and enjoys a general steadiness of weather to which the best climates on the continent of Europe are strangers. Fog is never seen. This island is almost exempt from the diseases peculiar to warm climates, and little subject to many of those which are common in more northerly countries. "With respect to the prevalence of consumption among the natives of Madeira, there is a difference of opinion among those who have had the best opportunities of observing." Experience shows that *confirmed* consumption is not benefited by a residence at Madeira; but "the effects of the climate on incipient cases, and those threatened with the disease from hereditary or acquired predisposition, are highly encouraging, and should lead medical men to recommend such a measure at the only time when it promises benefit." Invalids who intend to pass the winter in Madeira should leave this country in the end of September, or the beginning of October. The beginning of June is sufficiently early to leave the island to return to England.

In the second part of the work, the author first states the degree of benefit to be expected from climate in various diseases, and gives some excellent directions and cautions to travelling invalids. Disorders of the digestive organs and consumption are next commented upon, and the particular cases pointed out which are most likely to be relieved by change of climate. In chronic diseases of the larynx, trachea, and bronchia, if proper discrimination be exerted, much benefit may be expected from change of air and climate.

"Before the patient leaves his home, we ought to be assured that all acute and even subacute inflammation has ceased, or otherwise such a measure is more likely to increase than to diminish the disease. This is well exemplified in the effects of change of air in common catarrhal affections. A journey in the commencement of a cold generally increases it: if, on the contrary, the acute period of the cold has passed by, a short journey is one of the most effectual means of removing the cough entirely; and the same thing has been long observed in hooping cough." (P. 278.)

In conclusion, Dr. Clark makes a few brief remarks upon asthma, gout, and chronic rheumatism, which are chiefly in reference to the immediate purpose of his work.

A series of very useful and comprehensive meteorological tables is contained in the Appendix.

It would be impossible to select a subject upon which more erroneous opinions have been formed, both by the public and the profession, than that respecting the influence of the climate in different places, both at home and abroad, over the progress of various diseases. But few persons, comparatively, have had personal experience of the advantages which particular situations offer to the invalid; and, until the publication of Dr. Clark's work, the untravelled inquirer would have sought in vain for a general and trustworthy guide. The physician will find this work of the greatest utility to him as a book of reference: it will enable him to direct others with judgment, and the invalid who ventures out of medical leading-strings may confidently rely upon the information and impartiality of Dr. Clark. He is not a resident practitioner at either of the places he recommends, and therefore *his* book cannot be looked upon as a mere invitation to entice patients within his own focus.

*On a Morbid Affection of Infancy, arising from Circumstances of Exhaustion, but resembling Hydrencephalus.* By Marshall Hall, M.D. F.R.S.E. &c. &c.—8vo. pp. 40. Seeley, London, 1829.

Upon several occasions we have declared it to be our opinion that one of the most serious and most frequent practical errors of the present day, is the readiness with which many practitioners assume the existence of cerebral disease, and particularly of "water in the head," from the occurrence of various symptoms during infancy, which are by no means necessarily connected with any affection of the brain. The brief, but valuable, little Essay before us contains additional proofs of the danger that frequently exists of mistaking other, and very different, diseases of infants for hydrencephalus, and of consequently instituting a very improper, and probably destructive, mode of practice.

Dr. Hall has watched with peculiar care many cases of a morbid affection incident to infancy, which generally arises from circumstances of exhaustion, but resembles, in many of its symptoms, the earlier, and especially the later, stages of hydrencephalus; and, as this affection has not been noticed by practical writers as it deserves, he thinks the present brief account of it cannot prove uninteresting to the profession.

Dr. Hall first gave a cursory sketch of this morbid affection in his "Medical Essays," published in 1825, but which work is now out of print. It has since been briefly noticed by Dr. Abercrombie, in his "Researches on Diseases of the Brain and Spinal Cord." Dr. Gooch has also treated of this affection, in his recent "Account of some Diseases peculiar to Women:" and these are all the notices he has seen of this singular and interesting disorder.

Those diseases of children are best understood which arise from irritation in the stomach and bowels, and the irritation of teething and inflammation.

"But there is another source of disorder in infancy, less frequent perhaps in its operation, but not less important in its consequences, and far less understood by medical men, in exhaustion. This exhaustion has its origin in early infancy, chiefly in diarrhœa or catharsis; in the later periods of infancy, in the loss of blood, with or without the relaxed or evacuated condition of the bowels.

"The state of diarrhœa has generally depended upon improper food. It has very frequently succeeded to weaning, or to other changes in the diet. The catharsis has followed the administration of an aperient medicine, which, at such a moment of disorder of the stomach and bowels, is apt to act excessively. The exhaustion from loss of blood generally follows the inappropriate or undue application of leeches, or the use of the lancet.

"I may observe, indeed, in this place, that of the whole number of fatal cases of disease in infancy, a great proportion occur from this inappropriate or undue application of exhausting remedies. This observation may have a salutary effect in checking the ardor of many young practitioners, who are apt to think that, if they have only bled and purged, and given calomel enough, they have done their duty; when, in fact, in subduing a former, they have excited a new disease, which they have not understood, and which has led to the fatal result.

"This question, and that of the effects of exhaustion in infants and children, open a new field for investigation. Almost all our works on infantile diseases are silent on the subject; and yet, without an accurate knowledge of it, I regard it as totally impossible that we should be prepared to watch and treat the morbid affections of this young and tender age. The subject must be taken up and investigated anew. All the affections which may arise from exhaustion must be accurately observed, distinguished from similar affections arising from other causes, and traced back to their origin, and forward in relation to their remedies. In this manner some hydrencephaloid, convulsive, and even croupy affections, will be viewed in a new aspect; and we shall be preserved from some painful dilemmas into which we should assuredly fall without this knowledge of the effects of exhaustion." (P. 6.)

As in this essay Dr. Hall proposes to confine his observations to one of the forms of disorder which arise from this cause, the hydrencephaloid, it is not for us to travel out of the subject. We may just observe, however, in reference to the last passage in the above extract, that we have elsewhere laid especial stress upon the frequent occurrence of convulsive affections in infants, from various debilitating causes, and, amongst others, the excessive action of purgative medicines. We may, perhaps, venture to give the following passage: "There are doubtless cases in which it may be necessary for us to act freely and frequently upon the bowels of children. Let us, however, beware that we do not commit the common, but important, error of considering the general irritability which is induced by purgatives when long employed, as a state which demands their still further use, or of regarding the unusual appearance of the stools, which is dependent entirely upon their action, as a proof that the stomach and bowels are yet in a state of derangement, which is only to be relieved by further purgation."*

* Practical Observations on the Convulsions of Infants, by JOHN NORTH, Surgeon-Accoucheur. 1826. P. 177.

Dr. Whitlock Nicholl has adverted to this subject. He observes, that he has seen the erethismal state of the brain, which is so frequently the cause of convulsions, kept up, if not induced, by powerful purgatives, which in common practice are repeatedly given unnecessarily.

It is too much the fashion of the day to consider either local or general bleeding necessary in all convulsive affections of infants, although there can be no doubt that convulsions often occur in children of enfeebled constitutions, merely from want of sufficient nourishment, and that in such cases a light, yet generous, diet will be the most efficacious remedy. It was said, indeed, by Haller, and the doctrine has been adopted and repeated by Bichat, "that the vital force manifests itself in two opposite states, in paralysis and convulsions. The first is the sign of *diminished energy*, and the second of *augmented energy*." This assumption will assuredly not admit of general application, and, if indiscriminately acted upon, must be followed by injudicious practice. It is worthy of remark, that every animal which dies from *loss of blood* is attacked with violent convulsions during the last moments of its existence. How frequently also are puerperal women convulsed who have had considerable uterine hemorrhage. In such cases there can surely be no "augmented energy of the vital force;" for convulsions occur in these instances before any reaction takes place in the system which has been weakened by excessive bleeding.

The morbid affection which it is the object of the present essay to describe, may be divided into four stages:

"The first that of irritability, the second that of torpor: in the former there appears to be a feeble attempt at reaction, in the latter the nervous powers appear to be more prostrate. These two stages resemble, in many of their symptoms, the first and second stages of hydrencephalus respectively.

"This morbid affection has, as I have stated, usually been first induced by some change in the diet, by which the stomach has been loaded or disordered, and the bowels perhaps affected with diarrhœa; and this latter state has frequently been exasperated by the untimely administration of an aperient medicine. The infant becomes irritable, restless, and feverish; the face flushed, the surface hot, and the pulse frequent; there is an undue sensitiveness of the nerves of feeling, and the little patient starts on being touched, or from any sudden noise; there are sighing, moaning during the sleep, and screaming; the bowels are flatulent and loose, and the evacuations are mucous and disordered.

"If, through an erroneous notion as to the nature of this affection, nourishment and cordials be not given; or if the diarrhœa continue, either spontaneously or from the administration of medicine, the exhaustion which ensues is apt to lead to a very different train of symptoms. The countenance becomes pale, and the cheeks cool or cold; the eyelids are half closed, the eyes are unfixed, and unattracted by any object placed before them, the pupils unmoved on the approach of light; the breathing, from being quick, becomes irregular and affected by sighs; the voice becomes husky, and there is sometimes a husky teazing cough; and eventually, if the strength of the little patient continue to decline, there is crepitus or rattling in the breathing; the evacuations are usually green; the feet are apt to be cold.

"A similar train of symptoms occurs in other cases, in which the strength of the little patient has been subdued, and the vascular system exhausted, by the abstraction of blood. In both cases leeches are sometimes again applied to subdue this new form of disease, under the erroneous notion of a primary cerebral affection. This measure infallibly plunges the little patient into imminent, if not irretrievable, danger.

"Sometimes the sinking state goes on in spite of every appropriate remedy.

"Stimuli, if efficacious, reduce the frequency of the pulse, and restore the wonted warmth, colour, expression, and smiles to the countenance.

"The condition of the cheeks, in regard to colour and warmth, may be considered as the pulse of very young infants, indicating the degree of remaining power, or of exhaustion. In the present case especially, there is no symptom so important, so distinctive. It is from the condition of the cheeks, in conjunction with a due consideration of the history, that the diagnosis of this morbid state, and the indication of the appropriate remedies, are chiefly to be deduced. The general surface, and especially the hands and feet, also afford important sources of information as to the condition of the nervous or vital powers. Next to these, the degree of frequency of the pulse and the character of the breathing are points of the greatest importance. During the stage of irritability, the breathing is quick; during that of torpor, it is slower, irregular, suspirious, and finally crepitous; the pulse changes in its beat, from being full becoming smaller, but retaining, perhaps, its former frequency." (P. 8.)

We are to be especially careful not to mistake the stupor or coma into which the state of irritability is apt to subside, for the natural sleep, and for an indication of returning health.

"The pallor and coldness of the cheeks, the half-closed eyelid, and the irregular breathing, will sufficiently distinguish the two cases. It is equally important to distinguish this state from a hydrencephaloid affection arising from derangement of the alimentary canal, and from the coma of hydrencephalus itself. This is to be done chiefly by observing the condition of the countenance, and by tracing the history and causes of the affection. There is an absence of the heat and occasional restlessness and irritability of the former of these affections, and of the contracted brow, and of the expression of pain on moving the head, observed in the latter." (P. 11.)

Dr. Hall has been frequently consulted when the original disease has been subdued, perhaps, and when the chief complaint of the little sufferer was a state of exhaustion; which a truce from remedies and medicines, and a proper supply of nourishment, and perhaps stimulus, have removed.

"This state of things is often mistaken for inflammation of the brain, or hydrencephalus; and it may be difficult to state the grounds for a just diagnosis between the two affections. It will, however, be of great assistance to be fully aware of the nature and character of exhaustion, and to conjoin with this knowledge a due retrospect of the history of the case, and a due consideration of the effects of the various remedies which may have been employed." (P. 12)

Dr. Abercrombie also observes,* that, in the last stage of diseases of exhaustion, patients frequently fall into a state resembling coma a considerable time before death, and while the pulse can still be felt distinctly. He has many times seen children lie for a day or two in this kind of stupor, and recover under the use of wine and nourishment. "It is often," he says, "scarcely to be distinguished from the coma which accompanies diseases of the brain." It attacks them after some continuance of exhausting diseases, such as tedious and neglected diarrhœa.

Dr. Gooch observes,

"I am anxious to call the attention of medical men to a disorder of children which I find invariably attributed to, and treated as, congestion or inflammation of the brain, but which I am convinced often depends on, or is connected with, the opposite state of circulation. It is chiefly indicated by heaviness of head and drowsiness. The age of the little patients whom I have seen in this state has been from a few months to two or three years; they have been rather small of their age, and of delicate health, or they have been exposed to debilitating causes. The physician finds the child lying on its nurse's lap, unable or unwilling to raise its head, half asleep, one moment opening its eyes, and the next closing them again with a remarkable expression of languor. The tongue is slightly white; the skin is not hot, at times the nurse remarks that it is colder than natural; in some cases there is at times a slight and transient flush. The bowels I have always seen already disturbed by purgatives, so that I can scarcely say what

* On Diseases of the Brain, p. 310.

they are when left to themselves. Thus the state which I am describing is marked by heaviness of the head and drowsiness, without any signs of pain, great languor, and a total absence of all active febrile symptoms. The cases which I have seen have been invariably attributed to congestion of the brain, and the remedies employed have been leeches and cold lotions to the head, and purgatives, especially calomel. Under this treatment they have gradually become worse, the languor has increased, the deficiency of heat has become greater and more permanent, the pulse quicker and weaker, and at the end of a few days, or a week, or sometimes longer, the little patients have died with symptoms apparently of exhaustion. In two cases, however, I have seen, during the last few hours, symptoms of oppressed brain, as coma, stertorous breathing, and dilated and motionless pupil."* (P. 16.)

Dr. Hall now proceeds to state the remedies for this morbid affection. Diarrhœa is to be checked, the bowels afterwards regulated, and the strength of the child to be sustained and restored.

"With the first objects it may be necessary to give the tinctura opii and chalk, and afterwards the pilula hydrargyri, rhubarb, and magnesia; with the second, sal volatile, but especially brandy, and proper nourishment are to be given according to circumstances. But in this, as in so many cases of infantile disorders, the young milk of a young and healthy nurse, is the remedy of most importance; in the absence of which, ass's milk may be tried, but certainly not with the same confident hope of benefit.

"Five or ten drops of the sal volatile may be given every three or four hours; and, twice or thrice in the interval, five or ten drops of brandy may be given in arrowroot done in water. As the diarrhœa and the appearances of exhaustion subside, these remedies are to be subtracted; the bowels are to be watched and regulated, and the strength is to be continually sustained by the nurse's or ass's milk. The brandy has sometimes appeared to induce pain: sal volatile is then to be substituted for it; a dose of magnesia has also appeared to do good." (P. 18.)

For the state of irritability the warm bath will be proper. In every case the extremities are to be kept warm by flannel, and the circulation is then promoted by friction.

To exemplify the description of this "morbid affection," and its appropriate treatment, several cases are adduced. We give the following as an example:

"On Saturday, the 21st of March, I was called to an infant three months old, under the following circumstances: It had been weaned a fortnight; during this period it had been fed with milk and barley-water, and once a day with the addition of bread. It

* Account of some of the most important Diseases peculiar to Women. Pp. 357-358.

]

remained well until the Thursday before my visit, when it became affected with fever, restlessness, crying, and moaning in its sleep, and with diarrhœa, passing several undigested and mucous stools. A dose of calomel was given, which induced sickness. A second dose was then administered, which, in the course of that and the succeeding day (Friday), was followed by sixteen evacuations.

"During Friday night there were much heat, interrupted sleep, and griping pains, followed by offensive evacuations. On the following morning there was some degree of dozing or coma; the eyes were imperfectly closed, the tunica albuginea alone being visible; and the mouth was open. This inanimate state, attended by coldness of the cheeks, hands, and feet, would continue for ten minutes, and then there would be some degree of reaction.

"This state of things continued during the whole of Saturday, the dozing assuming the character of more settled coma. I saw the little patient late in the evening: the cheeks were then pale and cold; the eyes were half open and unfixed, and unexcited by any external object, however brilliant, and the pupils were moderately dilated, and unmoved on the approach of light; the pulse was 132; the breathing irregular and sighing; the general surface pale, and the hands and feet cold.

"There were thus the usual state of the comatose stage of hydrencephalus. The condition of the countenance, general surface, and extremities, and the history of the case, however, led me to view it as one of exhaustion, and not of inflammation and effusion within the head. I therefore prescribed five drops of brandy, and three of sal volatile, to be given alternately every hour; and I directed the little patient to be put, once in the interval of the two hours, to the breast of a young and healthy nurse.

"Under this discipline there was a gradual, but not unchequered amendment. The stupor began to alternate with restlessness, and there were frequent startings: more than once the restlessness was so great as to require the use of a warm bath, by which it was greatly relieved, and quiet and sleep induced. The countenance gradually assumed a more natural and animated appearance and expression, with an occasional smile. The bowels were moved four times on the succeeding day, the evacuations being green.

"On Monday morning a little magnesia and rhubarb were given, the other remedies having been, and being still, continued. The little patient started much less on this day, and slept quietly, and there was no return of restlessness to require the warm bath.

"On the succeeding days there was an obvious and progressive amendment. The brandy and sal volatile were gradually abstracted, the breast being continued." (P. 21.)

In another case Dr. Hall was requested to see a little girl, aged two years and three quarters, who had laboured under an attack of influenza. The affection of the chest had been severe and protracted, and sixteen leeches had been applied, besides the administration of other depletory measures, before it had subsided.

"The symptoms of affection of the chest were, however, subdued at last; but the little patient was left extremely exhausted, and in this state a new train of symptoms supervened, not less alarming, and more puzzling, than the first. The child fell into a dozing state, and lay with its eyelids but half closed; it moaned when any attempt was made to rouse it; the eyes were unfixed on any external object, the pupils were dilated, yet partially contractile on the influx of light; the pulse was 140.

"On withdrawing into an adjoining room, the medical gentleman whom I had the pleasure of meeting observed, "Hydrencephelas has now supervened, and we must administer calomel." I replied that I took a different view of the case; that it resembled hydrencephalus indeed, but arose from exhaustion; and that brandy, not calomel, could alone save the little patient's life. I referred to the history of the case for sufficient sources of exhaustion; and to the facts detailed in the preceding part of this paper for the actual occurrence of such cases in practice.

"We administered brandy, directing thirty drops to be given every two hours, with barley-water in the intervals, and a quarter of a pint of ass's milk twice in the twenty-four hours. The bowels were relieved by.magnesia and the warm-water injection.

"This plan of treatment lowered the number of the pulse, and gradually diminished the severity of the other symptoms. Still the eyes were not to be fixed by presenting any bright object before them; the pupils remained dilated; the tunica conjunctivæ became inflamed from exposure, between the partially closed eyelids; and once or twice the fæces were passed involuntarily in bed.

"The brandy having occasioned pain in the bowels, (an effect which I have several times observed,) it was given alternately with the spiritus ammoniæ aromaticus. The rest of the plan was pursued with unexampled assiduity by a most tender mother, who did not once undress or leave her little patient until she saw it out of all danger. This task was the severer because, although the symptoms which have been detailed subsided gradually and favorably, they were succeeded by an equally severe and sadly protracted aphthous affection.

"The first symptom of amendment was a diminished frequency of the pulse; the next a restored susceptibility of the pupils to light; then the eyes became attracted and fixed by external objects, and a smile began to play upon the little patient's countenance; the eyelids closed more and more perfectly during sleep, and the conjunctivæ lost their inflamed injected appearance; the knees were drawn up, and the posture on the side began to be assumed spontaneously." (P. 24.)

Cases of a similar nature have also been related to the author by Dr. TWEEDIE, Mr. HEMING, and other practitioners.

Since Dr. Hall's paper was read at the Medico-Chirurgical Society, Dr. Gooch has given us additional proofs of the frequency of such cases, in his recently published work.

This little essay is especially worthy the attention of junior practitioners, who are certainly too much in the habit of referring, without discrimination, all diseases of infants to inflammatory action, and of presuming that, whenever there are any symptoms of heaviness or drowsiness in a child, free bleeding and "heroic" doses of calomel are instantly demanded, to prevent effusion of water within the brain, or to remedy cerebral congestion. That cases of this sort do occur, and frequently too, there can be no doubt; but it is of the utmost importance that the practitioner should be aware of the occasional existence of a train of symptoms arising from *exhaustion*, which, without due attention, may be easily mistaken from hydrencephalus, when in fact there is no cerebral affection, and the infant consequently be bled or purged to death at the very moment when its safety depends upon a directly opposite mode of treatment. The diagnosis between these cases may, it is true, be somewhat difficult. It cannot be established by the presence or absence of any particular symptom; but it may from a careful consideration of the commencement, progress, and duration of each individual case. The *effect* of the treatment that is adopted will, of course, guide our opinion of the nature of the case. If symptoms be relieved by nourishment and moderate stimuli, it cannot be necessary to bleed or "push the calomel."

Dr. Marshall Hall deserves the best thanks of the profession for having first directed their attention to a subject which has very generally been overlooked by practitioners, notwithstanding its great practical importance.

---

*Elements of Practical Midwifery; or, a Companion to the Lying-in Room.* By Charles Waller, Consulting Accoucheur to the London and Southwark Midwifery Institution; and Lecturer on Midwifery and the Diseases of Women and Children, at the Medical School, Aldersgate street.—18mo. pp. 135. Highley, London. 1829.

The intention of this little volume is to present to the student of midwifery, in a condensed form, those rules which are particularly applicable to the practical department of the science.

The author trusts that the frequent inquiries amongst his own pupils for a concise book of reference, will sufficiently apologize for the present undertaking, which is intended as a remembrancer in the lying-in room, and consequently will by no means supersede the necessity of consulting the more voluminous treatises on obstetric science. To the gentlemen attending Mr. Waller's lectures it will certainly be useful, as it forms a syllabus of that part of the course in which the varieties of parturition are described.

The anatomical description of the pelvis is very briefly given, as it is presumed that those who are about to enter upon the practical part of midwifery, have previously made themselves acquainted with the structure of the parts concerned in parturition. Under the head of "Duties of the Accoucheur," much concise, yet very judicious advice, is given, which should be thoroughly impressed upon the mind of every student and young practitioner, who ought never to forget that his professional success will depend, in a great measure, upon his address and general conduct in the lying-in room. It could not be the intention of the author, in a work like the present, to enter upon the description of cases of very rare occurrence. In addition to the very proper remarks he has offered upon the management of the placenta, we may refer to a circumstance which would certainly perplex the novice in midwifery, and perhaps embarrass the senior practitioner.

It has occasionally happened, that, although the placenta *can* be felt at the upper part of the vagina, it is *not* perfectly detached from the uterus. Two instances of this kind have happened within our own knowledge, and the following case, which we extract from a very useful work,* conveys a good lesson upon the subject. A woman had been delivered without difficulty about three hours before the attendance of Dr. Ramsbotham, but the placenta remained behind, with flooding and fainting. She was much depressed, with a small weak pulse. The *greater part* of the placenta was down *in the vagina*, at which the midwife had been lugging, till she had nearly separated the funis; the *remainder* was in the uterus. On passing his hand, Dr. R. found that nearly one third of the mass was morbidly adherent to the fundus uteri; and, during its separation, the woman suffered an additional loss, so that, on withdrawing his hand, she fainted. The uterus contracted well, and there was no more hemorrhage. After being in a state of much depression and uncertainty for a few days,

* Practical Observations in Midwifery, by J. Ramsbotham, M.D., 1821, p. 105.

the patient recovered. That such cases are very rare, we admit, but they are much too important to be forgotten by the practical accoucheur.

As Mr. Waller has successfully practised the operation of transfusion, in cases of sinking and approaching death, after severe uterine hemorrhage, and also been very conspicuous for the zeal with which he has recommended this, we fear, still too much neglected practice, we shall extract his observation upon this highly important subject.

"So simple is the principle, so easy the performance, and so splendid have been the results of this operation, that it has borne down the clamour of its opponents, and may now fairly be said to be fixed upon as firm a basis as most other operations in surgery. The design of this work being to convey practical information, it is not intended to enter into any lengthened historical detail. As, however, the trials which were formerly made have been brought forward in evidence against transfusion, it will be but right to state that, as at present practised, it differs very materially both in principle and mode of performance.

"It was formerly recommended in certain diseased states of the constitution; and the blood was taken from the inferior animals, (the calf, sheep, &c.) It is now used as a remedy in desperate cases of hemorrhage; human, and not brute, blood being employed. This difference, though a very important one, was entirely overlooked by the objectors to the operation. Again, it has been asserted by some that transfusion is wholly unnecessary, because, if the blood were arrested, the patient would invariably recover without it; and, if the hemorrhage continued, that it would be useless, as the blood injected into the arm would immediately pass out again at the uterine artery. Many well-authenticated cases, however, have shown that the first assertion is incorrect;* and, with regard to the second, it remains to be proved whether, under the circumstances of the case, the introduction of fresh, pure, and living blood, would not, by acting as a stimulus to the system, induce such a state of contraction in the muscular structure of the womb as would prevent any further effusion. This is thrown out as a mere conjecture, as it has not yet been employed with this intent, and consequently is unsupported by facts; but, upon reflection, it seems probable that such would be the effect: at any rate, the attempt would be perfectly justifiable in a case otherwise hopeless; and it should be particularly borne in mind that under no other circumstance has this operation been hitherto performed.

"For the suggestion of transfusion of blood as a remedy in these desperate cases of hemorrhage, the profession and the public at large are under deep and lasting obligations to Dr. James Blundell;

* One melancholy example of this kind came under the author's own observation; the poor woman living three hours after the hemorrhage ceased. This case led him to think seriously of transfusion.

and, although the proposal was treated with 'neglect, opposition, and ridicule,' still he was not to be deterred from his purpose till the remedy had experienced a fair trial; being convinced, from his numerous and well-conducted experiments upon the dog, that in this animal, at any rate, the injection of canine blood into the veins was not only practicable and safe, so far as the operation was concerned, but that it really was applied to the nourishment of the system, and consequently was something more than a mere stimulus to the heart's action. This fact being established with regard to the dog, it required no great stretch of the imagination to suppose that human blood, injected into human veins, might also be made subservient to the purposes of human circulation; and upon this principle, and grounded upon these facts, a trial of it was recommended.

"From its novelty, however, some time elapsed before it was put into practice; and it is productive of great satisfaction to the author when he reflects that the first successful operation of transfusion was performed on one of his patients by Dr. Blundell and himself. The female was an exceedingly delicate, weakly creature, who had lost a large quantity of blood very suddenly after parturition, and in whom the most powerful stimulants failed to procure more than temporary benefit.*

"Dr. Blundell, Mr. Doubleday, and others, have in several instances successfully employed transfusion, and, with the exception of one case out of about fourteen,† (where the operation has been properly and carefully performed,) there has been no recorded instance of failure. When it is considered that the cases were otherwise desperate, and that perhaps the mechanical means (from its being a new remedy) were defective, its value must be highly esteemed; and it may, perhaps, be reckoned among the greatest improvements, or at any rate the most valuable addition, which has of late years been made to the means of the accoucheur, and one which is in itself sufficient to hand down the name of its projector to posterity, as one of the greatest benefactors to *womankind.* Nor is it likely (the safety and utility of the operation being fully established,) that its beneficial effects will be confined to the female sex, as it is equally applicable to the male when sinking from large losses of blood, whether from accident or any other cause.

"*Method of performing the operation.* The transfusion of blood from one person into the veins of another may be effected in various ways. The syringe has hitherto been employed, and as it is very conveniently and safely performed by means of this instrument, the reader's attention will not be distracted by the relation of any other method. An improvement to the common syringe has been

* This took place in August 1825. For a detailed account of the case, see the medical journals of that period. The author has twice performed the operation since that time, and with the most perfect success.

† The *precise* number has escaped the author's memory.

made by Mr. Lloyd, an ingenious instrument-maker, residing in King street, Borough: to the barrel of this is appended a small funnel, by means of which contrivance the blood passes directly from the arm of the person supplying it into the syringe, without being obliged to be first received into another vessel: some little time is thus gained, which is an object of importance. A stopcock is also attached to it, by turning which the communication may be opened either with the funnel or with the extremity of the instrument, according as the blood is either being received into the syringe from the funnel above, or is being passed into the vein of the patient. The instrument is made of brass, and well lined with tin; and it is scarcely necessary to add, should be perfectly cleaned before it is used, and slightly warmed by passing tepid water several times through it, taking care not to use it too hot, as it would have a tendency to coagulate the serum of the blood.

"The basilic or the cephalic vein of the patient is to be laid bare to the extent of an inch or an inch and a half, taking care to divest it of its surrounding cellular membrane. A blunt-pointed bent probe, or a curved and blunt needle, is then to be passed under its lower extremity, in order that pressure may, if necessary, be made upon it with the finger, and the blood be prevented from oozing out; which, by obscuring the orifice, would be productive of difficulty and delay. An opening should be made into the vein large enough easily to admit the point of the tubule which is attached to the extremity of the syringe. This instrument is made to contain two ounces only, it appearing from previous experiments to be safer to inject a small quantity at a time.

"These preparatory steps having been taken, a very free incision is to be made into the arm of the person about to furnish the blood, so that it may pass in a full stream into the funnel, and be from thence absorbed into the syringe; the stopcock must then be turned, and the funnel removed. The next part of the operation consists in expelling any quantity of air that may be contained within the instrument: for this purpose it is to be placed vertically, the handle below, the point upwards; the piston being gradually pressed upwards, till about a teaspoonful of blood is expelled. The point of the finger being then placed over the nozzle, the horizontal direction is to be given to the instrument, which should be insinuated about half an inch within the vein, in the direction, of course, towards the heart, and the blood *very slowly* and cautiously injected. This is a point of great importance to be observed; for the heart's action is in these instances so weak, that a sudden influx of blood would, in all probability, at once overwhelm it, a fact witnessed by the author in the experiments upon the horse before alluded to. On removing the syringe from the vein, it should be instantly well washed out with cold water. Before repeating the injection it is better to wait for the space of four or five minutes to allow the blood time to circulate over the body; it may then be repeated in the same manner, the patient being narrowly watched with regard to the effect it has produced upon her.

"Eight, ten, or twelve ounces of blood may be thus injected; and it will seldom, if ever, be found necessary to exceed this latter quantity, even where the hemorrhage has been very profuse. The intention of the operation is not to restore the blood-vessels to the same degree of fulness as previously existed, but so far to add to the power of the system that the heart may be enabled to continue its contractions. It should be remembered that this organ (the heart) having been for some time acting on a greatly diminished supply of blood, is well prepared to receive the stimulus which an additional quantity would afford it, although small in comparison to that which has been lost. This circumstance is proved by the fact that the pulse evidently improves, sometimes after the first, but always after the second injection; and the effect is in general permanent, there being no recurrence of the syncope afterwards, which affords pretty satisfactory evidence that the injected blood does not act as a mere stimulus, but that it gives *power* to the system.

"When a sufficient quantity of blood has been introduced, the probe or needle is to be removed from the arm, the edges of the wound brought together by means of adhesive plaster, and over this a bandage loosely applied: in fact, it should be treated as a common incised wound."

We confess that in general we regard very short roads to professional instruction with more doubts of their safety and advantages. This little volume may however be fairly recommended to the attention of the obstetrical student. It contains, in a very small compass, a great deal of very useful elementary information.

