# Erratum

**Published:** 1829-10

**Authors:** 


ERRATUM. At page 351, line 15, for "hodrencephalus" read "hydrencephalus."

